# Coffee consumption and all-cause and cardiovascular mortality in older adults: should we consider cognitive function?

**DOI:** 10.3389/fnut.2023.1150992

**Published:** 2023-10-24

**Authors:** Fabin Lin, Yisen Shi, Xinyang Zou, Huaicheng Wang, Shibo Fu, Xuefei Wang, Zeqiang Yang, Guofa Cai, Guoen Cai, Xilin Wu

**Affiliations:** ^1^Department of Neurology, Center for Cognitive Neurology, Institute of Clinical Neurology, Fujian Medical University Union Hospital, Fuzhou, China; ^2^Fujian Institute of Geriatrics, Fujian Medical University Union Hospital, Fuzhou, China; ^3^Fujian Key Laboratory of Molecular Neurology, Fujian Medical University, Fuzhou, China; ^4^Department of Neurosurgery, Fujian Medical University Union Hospital, Fuzhou, China; ^5^School of Information Engineering, Guangdong University of Technology, Guangzhou, China

**Keywords:** coffee, decaffeinated coffee, caffeine, cognition, cardiovascular disease, survival

## Abstract

**Background:**

The association between coffee and mortality risk has been found in most previous studies, and recent studies have found an association between coffee consumption and cognition. However, there is still a lack of research exploring whether the association between coffee and mortality is influenced by cognitive function.

**Objective:**

The purpose of this study was to explore the association of coffee, caffeine intake in coffee and decaffeinated coffee with all-cause mortality and cardiovascular disease (CVD) mortality in older adults with different cognitive performances.

**Methods:**

The study was based on data from the National Health and Nutrition Examination Survey (NHANES) 2011–2014. Coffee and caffeine consumption data were obtained from two 24-h dietary recalls. Individual cognitive functions were assessed by CERAD-word learning test (CERAD-WLT), animal fluency test (AFT), and digit symbol substitution test (DSST). In addition, principal component analysis (PCA) was performed with the above test scores to create global cognitive score. The lowest quartile of scores was used to classify cognitive performance. Cox regression and restricted cubic spline (RCS) were applied to assess the relationship between coffee and caffeine consumption and mortality.

**Results:**

In the joint effects analysis, we found that those with cognitive impairment and who reported without drinking coffee had the highest risk of all-cause and cardiovascular mortality compared with others. In the analysis of population with cognitive impairment, for all-cause mortality, those who showed cognitive impairment in the AFT displayed a significant negative association between their total coffee consumption and mortality {T3 (HR [95% CI]), 0.495 [0.291–0.840], *p* = 0.021 (trend analysis)}. For DSST and global cognition, similar results were observed. Whereas for CERAD-WLT, restricted cubic spline (RCS) showed a “U-shaped” association between coffee consumption and mortality. For CVD mortality, a significant negative trend in coffee consumption and death was observed only in people with cognitive impairment in AFT or DSST. In addition, we observed that decaffeinated coffee was associated with reduced mortality in people with cognitive impairment.

**Conclusion:**

Our study suggested that the association between coffee consumption and mortality is influenced by cognition and varies with cognitive impairment in different cognitive domains.

## 1. Introduction

Coffee’s unique flavor and refreshing properties have made it one of the most popular beverages worldwide (1). Studies have shown that coffee may have an ameliorative effect on depression, cardiovascular disease and type 2 diabetes (2–4), and some studies have also revealed it can reduce the risk of all-cause mortality and cardiovascular mortality (5, 6). Moreover, several studies previously reported that coffee consumption might reduce the risk of developing cognitive impairment (7, 8).

Cognitive impairment is one major health issues facing older adults (9). According to studies, people with cognitive impairment tend to have a higher risk of developing Alzheimer’s dementia, one of the leading causes of death among older adults in the United States (10, 11). The WHO estimates that the prevalence of dementia will increase exponentially every 20 years and may exceed 115 million by 2050 (12). Besides, a growing number of studies show that older adults with cognitive impairment often suffer from a higher risk of death (13, 14). Therefore, the discovery of early preventive measures to reduce the risk of mortality in the cognitively impaired population would be of value to the older adults. In previous meta-analyses and multiple cohort studies, higher coffee and caffeine consumption has been found to be associated with a lower risk of all-cause mortality and cardiovascular mortality (5, 6). However, there is still a lack of research to confirm whether this association between coffee and death existed in people with cognitive impairment. To explore this association, we conducted a cohort study using data from the National Health and Nutrition Examination Survey (NHANES) from 2011 to 2014, applying three tests [the Consortium to Establish a Registry for Alzheimer’s Disease (CERAD) test, animal fluency test and digit symbol substitution test (DSST)] and principal component analysis (PCA) to define cognitive impairment, and obtained dates of all-cause and cardiovascular mortality by linking to the National Health Center (NHC) death files.

In this study, through analysis of a representative sample of older adults aged 60 years or older in the United States, the associations of total coffee consumption, caffeine intake from coffee, and decaffeinated coffee with all-cause mortality and cardiovascular mortality were explored separately for those who demonstrated cognitive impairment on three cognitive tests or global cognitive scores.

## 2. Materials and methods

### 2.1. Study design and population

The data analyzed in this study were obtained from two cycles of the National Health and Nutrition Examination Survey (NHANES) (2011–2012 and 2013–2014) and linked to public-use mortality files up to December 2019 (15). NHANES uses a complex, multistage, probability sampling design (16). Dietary data were collected by conducting home interviews and inviting participants to the mobile examination center (MEC) to complete questionnaires. The study was approved through the National Health Statistics Research Ethics Review Board, and informed consent was obtained from participants prior to data collection.

### 2.2. Cognitive performance assessment

Cognitive function was assessed by the Consortium to Establish a Registry for Alzheimer’s Disease (CERAD) word learning sub-test, animal fluency test (AFT), and digit symbol substitution test (DSST) (17). These tests were carried out in the mobile examination center (MEC). The CERAD word learning test (CERAD-WLT) assessed immediate and delayed learning of new language information and consisted of three consecutive learning trials, in which participants were asked to read aloud 10 unrelated words that appeared on a computer monitor, one at a time, and recall as many words as possible immediately after all words were presented. The order of these 10 words were different between each of the three trials. After participants completed the AFT and DSST, the delay recall test was administered (18). In the AFT, which was used to assess categorical verbal fluency, participants were asked to name as many animals as possible that came to mind within 1 min, and each animal named was given a score (19). The DSST, on the other hand, is a module of the Wechsler Adult Scale and is used to assess processing speed, sustained attention, and working memory (20). This test was conducted via a paper chart with a key at its top containing nine numbers and symbols. The participants were asked to fill in the 133 blocks adjacent to the numbers in the chart with matching symbols within 2 min, with the final total number of correct matches being the score on the DSST. For the assessment of global cognition, the three cognitive measures were entered into principal component analysis (PCA), and scores on the first unrotated principal component were saved, where higher scores represented better cognitive abilities. The specific analysis results about PCA are shown in Supplementary Table 1 and Supplementary Figure 1. Consistent with previous related studies (21–23), we used the 25th percentile (lowest quartile) of the scores in the three tests (CERAD-WLT, AFT, and DSST) as the cut-off point for determining cognitive impairment; the participants who scored within this quartile were categorized as having cognitive impairment, while those who scored above this threshold were categorized as having normal cognitive function. The cut-off points of CERAD test, AFT, DSST and global cognition were 21, 13, 34 and −0.98, respectively.

### 2.3. Dietary intake assessment

Data of coffee consumption, as well as caffeine intake from coffee and total energy intake, were obtained from two 24-h dietary recall interviews conducted by NHANES. The first interview was conducted in-person in the MEC, while the second interview was conducted by phone 3–10 days later after the first interview. During the MEC interview, the interviewers adopted a set of measurement guides (various glasses, bowls, mugs, bottles, household spoons, measuring cups and spoons, rulers, thick and thin sticks, bean bags, and circles, etc.) for participants to use in reporting food consumption, and after completing the MEC interview, participants were given a food model booklet to use in reporting food amounts at the phone follow-up. The NHANES individual food file, (24, 25) which contained a list of food and beverage consumption that was provided for each participant, and a “USDA (the US Department of Agriculture) food code” consisting of eight numbers was provided to identify these foods and beverages. A food description file corresponding to the code was provided in the USDA’s Food and Nutrient Database for Dietary Studies (FNDDS). According to the FNDDS, the USDA food code starting with “921” matches the coffee beverage in the two cycles of 2011–2012 and 2013–2014. Based on the USDA food code, we calculated the total coffee beverage consumption and caffeine intake form coffee of each participant. In addition, based on the food descriptions provided by the FNDDS, we further calculated the consumption of decaffeinated coffee and caffeine intake from coffee for each participant. The total energy intake for each participant was calculated from the NHANES total nutrient intake file (26, 27). For the participants who provided comprehensive data from two dietary recalls, we averaged the data from both recalls, while only the data from first recall was used for the participants who only conducted the first dietary recall.

To explore the associations between coffee consumption and mortality more thoroughly, we categorized each type of coffee consumption. For total coffee consumption, we categorized those with no coffee intake into a separate group, and then categorized the coffee consumption into three separate groups depending on the amount of coffee consumed, resulting in four total groups: (1) without coffee consumption; (2) 0.1–262.5 g/day (T1); (3) 262.6–496 g/day (T2); and (4) > 496 g/day (T3). The sample population that did not consume decaffeinated coffee (*n* = 2,156) was large. Therefore, the decaffeinated coffee consumption population was divided into three groups: (1) no coffee consumption; (2) only decaffeinated coffee consumption; and (3) others. The caffeine intake from coffee was grouped using the same criteria as how the total coffee consumption was grouped: (1) without caffeine intake, (2) 0.1–76 mg/day (T1), (3) 76.1–171.5 mg/day (T2), and (4) > 171.5 mg/day (T3).

### 2.4. Mortality

In this study, the primary outcome was all-cause mortality, while the secondary outcomes were cardiovascular disease (CVD) mortality. The 2019 open-access mortality file provided by the National Health Center (NHC) provided mortality follow-up data of the population included in this study (15), with the follow-up time defined as the number of months from the date access examination at the MEC to either the date of death or the end of the death follow-up period (31 December 2019), whichever occurred first. The specific causes of death in the mortality file were coded according to the international classification of diseases-10 (ICD-10), in which heart disease was identified as I00-I09, I11, I13, and I20-I51, while I60-I69 represented death from cerebrovascular disease. We used the codes corresponding to heart disease and cerebrovascular disease deaths to identify CVD mortality.

### 2.5. Covariates

Based on previous studies, a series of covariates was selected for the follow-up study (28–30). The sociodemographic factors included: age, gender, race (Mexican American, other Hispanic, Non-Hispanic White Person, Non-Hispanic Black Person, and other races), poverty income ratio (PIR) (≤ 0.99 or > 0.99), and educational level (less than 9th grade, 9–11th grade, high school graduate/GED or equivalent, some college or AA degree, and college graduate or above). The health-related lifestyle factors included smoking status (never: smoked less than 100 cigarettes in life; former: smoked more than 100 cigarettes in life and smoke not at all now; now: smoke more than 100 cigarettes in life and smoke some days or every day) and drinking status (whether drink 12 alcoholic beverages per year or not).

Clinical variables included body mass index (BMI) (< 25 kg/m^2^, 25–30 kg/m^2^ and ≥ 30 kg/m^2^), diabetes, hypertension, chronic kidney disease (CKD), cardiovascular disease (CVD), and cancer. Diabetes was defined as meeting at least one of the following conditions: (1) self-reported a physician diagnosis of diabetes; (2) taking insulin or other prescription diabetes medication; (3) glycosylated hemoglobin (%) > 6.5%. Hypertension was noted if the participants met at least one of the following three criteria: (1) self-reported history of hypertension; (2) current use of antihypertensive medications; (3) average systolic blood pressure ≥ 140 mm Hg and/or average diastolic blood pressure ≥ 90 mm Hg. CKD was noted if eGFR < 60 mL/min/1.73 m^2^ or ACR (albumin: creatinine ratio) ≥ 30 mg/g; the eGFR was calculated using the chronic kidney disease epidemiology collaboration (CKD-EPI) equation (31). We had the participants self-report CVD if they suffered from one of the following 5 conditions: coronary heart disease, congestive heart failure, angina, heart attack, and stroke (32). In addition to the above variables, we also included the NHANES survey cycle and total dietary energy intake as covariates.

### 2.6. Statistical analysis

According to the NHANES analysis guidelines, we created a new sample weight by dividing the original sample weights in the two cycles by 2; this new sample weight was used for the following analysis. The normally distributed continuous variables were represented as the mean ± SD, the non-normally distributed continuous variables were represented as the median (IQR: interquartile range), and the categorical variables were represented as the weighted percentage.

To elucidate the relationships between coffee consumption and all-cause and cardiovascular disease (CVD) mortality in people with cognitive impairment, we first explore the joint effect of cognitive function and coffee consumption on mortality, we created four mutually exclusive risk groups: Group 1 for normal cognition and had coffee consumption; Group 2 for normal cognition and without coffee consumption; Group 3 for cognitive impairment and had coffee consumption; and Group 4 for cognitive impairment and without coffee consumption. Cox regression was used to further estimate the hazard ratios of the other three groups when using Group 1 as a reference. The results were represented by hazard ratios (HRs) and 95% confidence intervals (CI). We adjusted for none in crude model. For the multivariate adjustment model, we adjusted age, sex, race, pir, educational levels, smoking status, drinking status, NHANES survey cycle, BMI, total energy intake, hypertension, diabetes, CKD, CVD, and cancer. Considering that the effect of specific coffee consumption on mortality may vary among people with different types of cognitive impairment, we further explored the association between specific coffee (total/decaffeinated coffee) consumption and mortality in people with cognitive impairment using multivariate Cox regression. The trend test (P-trend) was also used to analyze the linear relationship between coffee consumption and mortality; the restricted cubic spline (RCS) was used to analyze the non-linear relationship. In addition, a series of analyses of caffeine intake in coffee were conducted to make the association between coffee consumption and mortality more reliable. We also conducted a series of sensitivity analyses to test the stability of the results. First, we conducted a replicate analysis using the unweighted sample. Second, considering that the joint effects of higher caffeine content coffee consumption and cognitive function may be different from the regular coffee, an additional analysis was performed. High caffeine coffee was judged by the amount of caffeine in coffee [caffeine content (mg) divided by total coffee mass (g)], using the third quartile of the amount of caffeine in coffee for all single coffees consumed as reported by NHANES participants from 2011 to 2014 as the cut-off point. Coffee above this cut-off point was defined as “higher caffeine content coffee.” Third, we classified coffee into two types: instant and non-instant coffee. Then, the joint effect of their consumption and cognitive function on mortality was explored separately.

For covariates identified as missing < 10% at baseline, including marital status (< 1%); PIR (7%); education level (< 1%); BMI (1%); smoking status (< 1%); and drink status (< 1%), multiple imputation was used to address. All statistical analyses were completed by R Project for Statistical Computing (version 4.2.0) and the Schoenfeld residuals in the Cox regression analysis did not indicate a deviation from the proportionality assumption (33). Two-sided *p* < 0.05 was considered statistically significant.

## 3. Results

### 3.1. Characteristics of the study population at baseline

**FIGURE 1:**
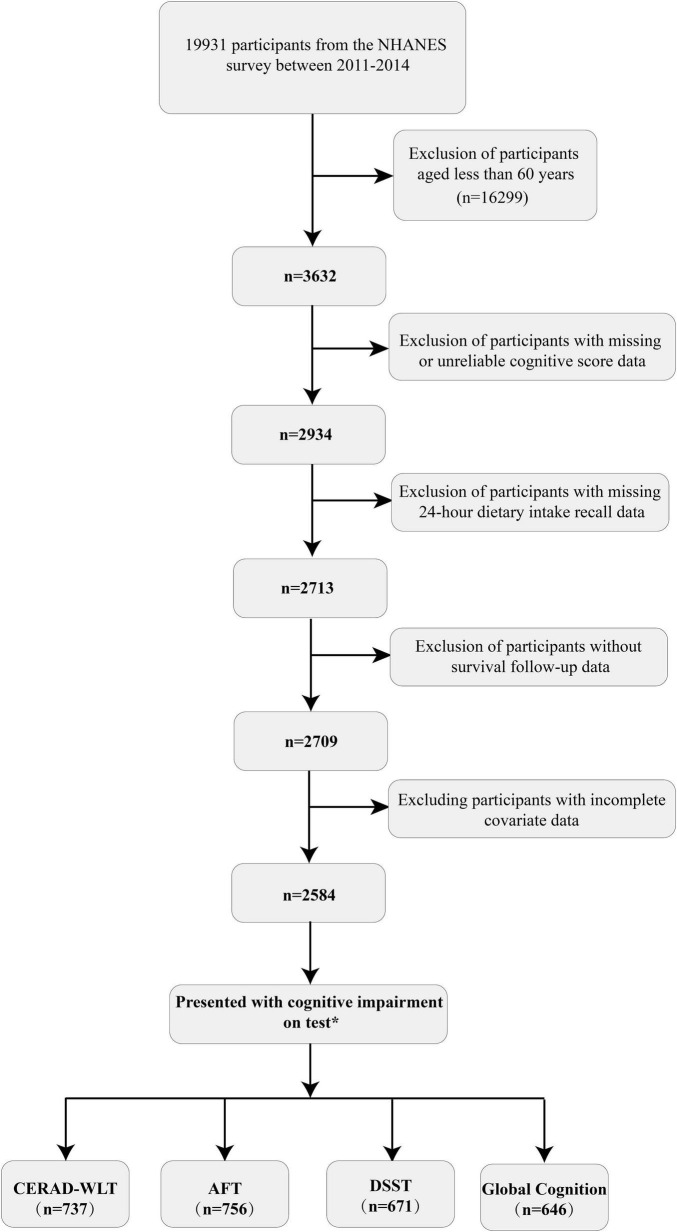
Flow chart of the selection process for selecting eligible participants.

In the population screening, we first included participants aged 60 or above with complete cognitive test data (*n* = 2,934) and then excluded those with missing dietary recall data and survival follow-up data (*n* = 225). We also excluded some individuals who had incomplete covariates (covariate with missing values excess 10%) data (*n* = 125). Ultimately, a total of 2,584 participants were included in this study, with a median follow-up time of 78 months for the included population. The specific screening process and number of people showing cognitive impairment on individual tests is represented in Figure 1. Characteristics such as lifestyle habits and disease history of the overall participants included and the four cognitive impairment groups obtained by different cognitive test scores are shown in detail in Table 1.

**TABLE 1:** Characteristics of study participants in NHANES 2011–2014 (*n* = 2,584).

Variable	Total	Cognitive impairment in CERAD-WLT	Cognitive impairment in AFT	Cognitive impairment in DSST	Cognitive impairment in global cognition
Age (year)^1^	68.00 (11.00)	73.00 (68.00, 80.00)	72.00 (14.00)	74.00 (13.00)	75.00 (12.00)
Total energy intake (kcal/day)^1^	1,801.00 (842.50)	1,712.50 (871.50)	1,614.50 (869.00)	1,538.00 (796.00)	1,559.00 (849.50)
**Caffeine intake from coffee (mg/day)^2^**					
0	747	220 (28.21)	226 (28.20)	212 (29.02)	206 (30.91)
0.1 to < 76	613	202 (23.43)	208 (23.30)	197 (25.21)	190 (25.60)
76.1 to < 171.5	617	172 (22.16)	188 (23.05)	151 (22.58)	147 (22.86)
> 171.5	607	143 (26.21)	134 (25.45)	111 (23.19)	103 (20.63)
**Total coffee intake (g/day)^2^**					
0	745	218 (28.10)	225 (28.15)	211 (28.93)	204 (30.76)
0.1 to < 262.5	615	203 (23.24)	226 (24.84)	195 (26.54)	190 (25.79)
262.6 to < 496	614	176 (22.00)	173 (21.75)	154 (20.81)	149 (21.28)
> 496	610	140 (26.66)	132 (25.26)	111 (23.72)	103 (22.18)
**Decaffeinated coffee consumption^2^**					
No coffee consumption	745	218 (28.10)	225 (28.15)	211 (28.93)	204 (30.76)
Only decaffeinated coffee consumed	260	87 (11.52)	84 (11.27)	76 (9.62)	84 (12.45)
Others	1,579	432 (60.37)	447 (60.58)	384 (61.45)	358 (56.79)
**Year cycle (%)^2^**					
2011–2012	1,195	400 (56.24)	356 (47.96)	328 (46.98)	330 (51.10)
2013–2014	1,389	337 (43.76)	400 (52.04)	343 (53.02)	316 (48.90)
**Gender (%)^2^**					
Male	1,282	458 (57.28)	367 (45.83)	381 (50.27)	371 (50.90)
Female	1,302	279 (42.72)	389 (54.17)	290 (49.73)	275 (49.10)
**Race (%)^2^**					
Mexican American	223	78 (4.88)	60 (4.27)	85 (8.10)	71 (6.70)
Other Hispanic	260	95 (5.83)	93 (6.29)	129 (12.02)	108 (10.04)
Non-Hispanic White Person	1,294	346 (75.50)	278 (66.78)	203 (57.37)	229 (61.89)
Non-Hispanic Black Person	586	168 (9.20)	238 (15.23)	224 (19.62)	198 (16.63)
Other race (Including multi-racial)	221	50 (4.59)	87 (7.44)	30 (2.89)	40 (4.74)
**Educational level (%)^2^**					
Less than 9th grade	282	166 (12.90)	138 (12.74)	219 (24.98)	183 (19.88)
9–11th grade (includes 12th grade with no diploma)	353	131 (15.73)	147 (16.04)	154 (21.60)	139 (18.24)
High school graduate/GED or equivalent	612	175 (26.08)	207 (30.46)	157 (26.37)	158 (29.70)
Some college or AA degree	735	150 (25.86)	169 (24.90)	97 (17.81)	103 (19.67)
College graduate or above	602	115 (19.43)	95 (15.86)	44 (9.24)	63 (12.51)
**Body mass index (%)^2^**					
< 25 kg/m2	683	202 (27.83)	218 (28.77)	180 (29.41)	170 (29.50)
25 to < 30 kg/m2	931	292 (40.19)	272 (37.00)	247 (34.69)	248 (38.42)
≥ 30 kg/m2	970	243 (31.98)	266 (34.23)	244 (35.89)	228 (32.09)
**Poverty–income ratio (%)^2^**					
≤ 0.99	386	148 (14.04)	163 (14.53)	186 (22.24)	157 (18.16)
≥ 1	2,198	589 (85.96)	593 (85.47)	485 (77.76)	489 (81.84)
**Hadat least 12 alcohol drinks per year (%)^2^**					
Yes	1,791	509 (68.19)	483 (64.28)	431 (61.25)	411 (59.92)
**Smoking status (%)^2^**					
Never	1,275	360 (51.42)	379 (50.46)	318 (49.13)	305 (48.75)
Former	986	280 (37.83)	276 (37.65)	243 (36.78)	247 (39.19)
Now	323	97 (10.75)	101 (11.90)	110 (14.10)	94 (12.06)
**Hypertension (%)^2^**					
Yes	1,840	552 (76.25)	592 (76.93)	532 (80.00)	504 (80.99)
**Diabetes (%)^2^**					
Yes	753	247 (26.63)	272 (31.11)	272 (36.73)	262 (36.26)
**CVD (%)^2^**					
Yes	589	229 (32.85)	190 (26.49)	192 (33.82)	201 (34.49)
**CKD (%)^2^**					
Yes	909	340 (46.79)	328 (44.32)	318 (52.17)	326 (55.14)
**Cancer (%)^2^**					
Yes	511	142 (22.92)	131 (21.09)	106 (21.15)	108 (21.22)
**Mortality status (%)^2^**					
Alive	2,089	509 (67.44)	550 (70.25)	463 (63.67)	435 (60.49)
Deceased	495	228 (32.56)	206 (29.75)	208 (36.33)	211 (39.51)
**Cardiovascular mortality (%)^2^**					
Yes	167	86 (11.99)	78 (11.28)	77 (13.56)	82 (15.72)

CERAD-WLT, Consortium to Establish a Registry for Alzheimer’s Disease Word Learning sub-test; AFT, animal fluency test; DSST, digit symbol substitution test; CVD, cardiovascular disease; CKD, chronic kidney disease.

^1^Data is represented as median (IOR).

^2^Data is represented as number of subjects (weighted percentages).

### 3.2. Joint effect on all-cause mortality and CVD mortality

Using a weighted Cox proportional hazard model, which adjusts for various confounding variables, to investigate the joint effect (Table 2), We found that the group with cognitive impairment (Group 3, 4) had a significantly higher all-cause and CVD mortality rate compared to Group 1 (*P* < 0.05). What’s more, we found that the group with cognitive impairment and without coffee consumption (Group 4) had the highest all-cause and CVD mortality among the four groups. However, we found that among the two groups (Group 1, 2) that both presented normal cognition, Group 2, the group with coffee consumption, did not display a significant increase in mortality compared to Group 1, as shown for both all-cause and cardiovascular mortality.

**TABLE 2:** The joint association of cognitive impairment and total coffee consumption with all-cause mortality and cardiovascular mortality.

	Cognitive performance (CERAD-WLT) combined with total coffee intake	Cognitive performance (AFT) combined with total coffee intake	Cognitive performance (DSST) combined with total coffee intake	Cognitive performance (global cognition) combined with total coffee intake
**All-cause mortality**				
**Crude HR (95% CI), *P-*value**				
Group 1	1 [Reference]	1 [Reference]	1 [Reference]	1 [Reference]
Group 2	1.021 (0.712, 1.464) 0.912	0.866 (0.617, 1.216) 0.407	0.876 (0.642, 1.195) 0.404	0.919 (0.636, 1.327) 0.653
Group 3	**2.810 (2.183, 3.618) < 0.001**	**2.190 (1.658, 2.894) < 0.0001**	**2.811 (2.018, 3.916) < 0.001**	**3.393 (2.509, 4.589) < 0.001**
Group 4	**2.877 (1.923, 4.303) < 0.001**	**3.034 (2.004, 4.591) < 0.0001**	**3.908 (2.625, 5.820) < 0.001**	**3.939 (2.775, 5.592) < 0.001**
**Multiple adjusted HR^1^ (95% CI), *P-*value**				
Group 1	1 [Reference]	1 [Reference]	1 [Reference]	1 [Reference]
Group 2	1.208 (0.829, 1.760) 0.325	1.109 (0.793–1.552) 0.546	1.111 (0.796–1.552) 0.536	1.135 (0.776, 1.661) 0.514
Group 3	**1.485 (1.112, 1.982) 0.007**	**1.445 (1.101–1.895) 0.008**	**1.606 (1.153–2.238) 0.005**	**1.629 (1.167, 2.275) 0.004**
Group 4	**2.185 (1.461, 3.267) < 0.001**	**2.490 (1.674–3.704) < 0.001**	**2.851 (1.906–4.264) < 0.001**	**2.657 (1.760, 4.013) < 0.001**
**Cardiovascular mortality**				
**Crude HR (95% CI), *P-*value**				
Group 1	1 [Reference]	1 [Reference]	1 [Reference]	1 [Reference]
Group 2	1.222 (0.740, 2.018) 0.433	1.039 (0.586, 1.842) 0.895	1.094 (0.649, 1.844) 0.736	1.063 (0.617, 1.829) 0.826
Group 3	**3.747 (2.710, 5.180) < 0.001**	**3.011 (2.036, 4.453) < 0.001**	**3.795 (2.180, 6.609) < 0.001**	**4.949 (3.387, 7.231) < 0.001**
Group 4	**4.131 (2.230, 7.651) < 0.001**	**4.192 (2.276, 7.720) < 0.001**	**4.984 (3.202, 7.756) < 0.001**	**6.037 (3.521, 10.350) < 0.001**
**Multiple adjusted HR^1^ (95% CI), *P-*value**				
Group 1	1 [Reference]	1 [Reference]	1 [Reference]	1 [Reference]
Group 2	1.358 (0.790, 2.336) 0.269	1.293 (0.732–2.284) 0.377	1.289 (0.734–2.263) 0.376	1.234 (0.705, 2.159) 0.462
Group 3	**1.734 (1.192, 2.522) 0.004**	**1.811 (1.199–2.735) 0.005**	1.618 (0.956–2.736) 0.073	**1.987 (1.335, 2.958) < 0.001**
Group 4	**2.727 (1.436, 5.179) 0.002**	**2.994 (1.458–6.145) 0.003**	**2.965 (1.817–4.837) < 0.0001**	**3.566 (1.943, 6.546) < 0.001**

Group1: Normal cognition + had coffee consumption;

Group2: Normal cognition + no coffee consumption;

Group3: Cognitive impairment + had coffee consumption; Group4: Cognitive impairment + no coffee consumption.

^1^Multivariable Cox proportional hazards models were adjusted for age, gender, race, PIR, educational levels, smoking status, drinking status, NHANES survey cycle, BMI, total energy intake, hypertension, diabetes, CKD, CVD, and cancer. Bold represents *P*-value < 0.05.

### 3.3. The association of coffee consumption with all-cause and CVD mortality among people with cognitive impairment

**FIGURE 2:**
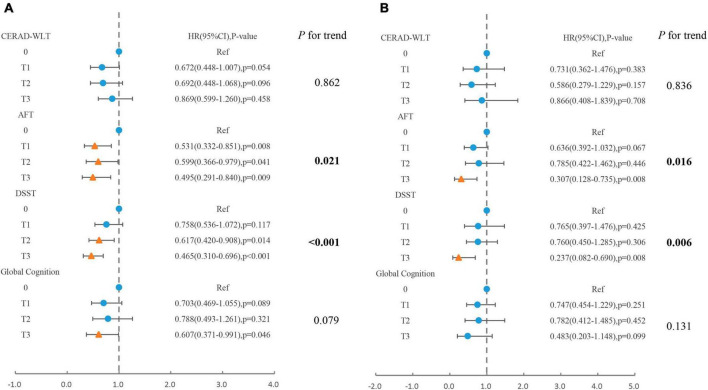
Association of total coffee consumption with **(A)** all-cause mortality, **(B)** cardiovascular mortality in older adults presenting with cognitive impairment in CERAD-WLT, AFT, DSST, or global cognition. The blue origin represents *P*-value > 0.05, the yellow triangle represents *P* < 0.05. T1: 0.1–262.5 g/day; T2: 262.6–496 g/day; T3: > 496 g/day. Model were adjusted for age, gender, race, PIR, educational levels, smoking status, drinking status, NHANES survey cycle, BMI, total energy intake, hypertension, diabetes, CKD, CVD, and cancer.

**FIGURE 3:**
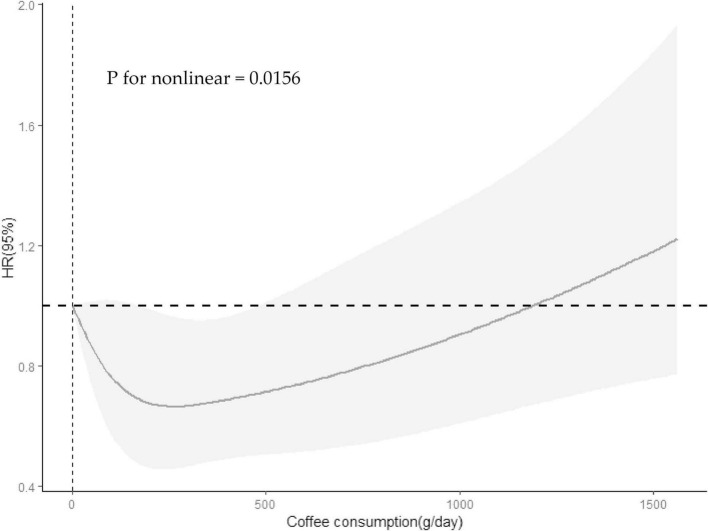
Restricted cubic spline revealed association between coffee consumption and all-cause mortality among participants with cognitive impairment in the CERAD-WLT. The Solid lines and shadows represent the estimated HRs and their 95% confidence intervals (HR, hazard ratio). Model were adjusted for age, gender, race, PIR, educational levels, smoking status, drinking status, NHANES survey cycle, BMI, total energy intake, hypertension, diabetes, CKD, CVD, and cancer.

The association between specific coffee consumption and all-cause mortality among older adults with cognitive impairment was presented in Figure 2. This regression adjusted for age, sex, race, PIR, educational levels, smoking status, drinking status, NHANES survey cycle, BMI, total energy intake, hypertension, diabetes, CKD, CVD, and cancer. For older adults with cognitive impairment in the CERAD-WLT, the regression model found a tendency for all-cause mortality to decrease only at T1 (HR = 0.67, 95% CI: 0.45–1.01, *P* = 0.054), and we further found a “U” shaped relationship between coffee consumption and all-cause mortality through the weighted RCS curve (*P* for non-linear = 0.016) (Figure 3).

In AFT or DSST presented with cognitive impairment, trend tests showed a linear trend between increased coffee consumption and decreased risk of all-cause mortality and cardiovascular mortality (*P* for trend < 0.05). However, for those who showed cognitive impairment in global cognition, the risk of all-cause mortality decreased significantly only when coffee consumption reached T3 (HR = 0.61, 95% CI: 0.37–0.99, *P* = 0.046).

### 3.4. The association of caffeine intake from coffee with all-cause and CVD mortality among people with cognitive impairment

**FIGURE 4:**
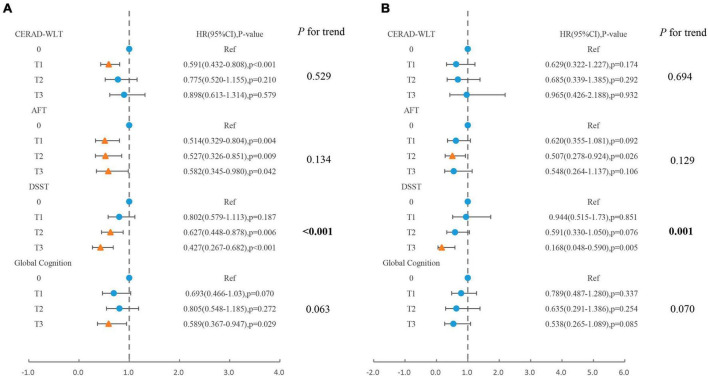
Association of caffeine intake from coffee with **(A)** all-cause mortality, **(B)** cardiovascular mortality in older adults presenting with cognitive impairment in CERAD-WLT, AFT, DSST, or global cognition. The blue origin represents *P*-value > 0.05, the yellow triangle represents *P* < 0.05 (T1: 0.1–76 mg/day; T2:76.1–171.5 mg/day; T3: > 171.5 mg/day). Model were adjusted for age, gender, race, PIR, educational levels, smoking status, drinking status, NHANES survey cycle, BMI, total energy intake, hypertension, diabetes, CKD, CVD, and cancer.

For populations that presented with cognitive impairment in the CERAD-WLT, the association between caffeine intake and all-cause mortality was observed to be similar to that of total coffee consumption, with a significantly lower risk of mortality for older adults whose caffeine intake was at T1 (HR = 0.59, 95% CI: 0.43–0.81, *P* < 0.001) (Figure 4).

For AFT, no significant linear trend was observed between caffeine intake and all-cause or cardiovascular mortality, and for all-cause mortality, the risk of death was slightly elevated when caffeine intake was at T3 (HR = 0.58) compared to T2 (HR = 0.53). In addition, the risk of cardiovascular death showed a significant decrease only in the T2 concentration interval (HR = 0.51, 95% CI: 0.28–0.92, *P* = 0.026).

For DSST, similar associations to coffee consumption were observed between caffeine intake and all-cause mortality or cardiovascular mortality, both indicating linear trends. For global cognition, caffeine intake was similar only when reaching T3 revealed a significant association with reduced risk of all-cause mortality.

### 3.5. The association of decaffeinated coffee consumption with all-cause and CVD mortality among people with cognitive impairment

For all-cause mortality, we found that for people with cognitive impairment, those who consumed only decaffeinated coffee showed a significantly lower risk of death compared to those who did not consume coffee (Table 3). For cardiovascular mortality, a significant reduction in mortality was found for those who consumed only decaffeinated in all three scores defined as cognitive impairment except for CERAD-WLT.

**TABLE 3:** Association of decaffeinated coffee consumption with all-cause mortality and cardiovascular mortality among older adults with cognitive impairment.

	Cognitive impairment presented in CERAD-WLT	Cognitive impairment presented in AFT	Cognitive impairment presented in DSST	Cognitive impairment presented in global cognition
**All-cause mortality**				
**Multiple adjusted HR^1^ (95% CI), *P*-value**				
None	1 [Reference]	1 [Reference]	1 [Reference]	1 [Reference]
Only intake decaffeinated coffee	**0.468 (0.267, 0.819) 0.008**	**0.385 (0.229, 0.648) < 0.001**	**0.539 (0.372, 0.780) 0.001**	**0.442 (0.265, 0.739) 0.002**
**Cardiovascular mortality**				
**Multiple adjusted HR^1^ (95% CI), *P-*value**				
None	1 [Reference]	1 [Reference]	1 [Reference]	1 [Reference]
Only intake decaffeinated coffee	0.545 (0.147, 2.011) 0.362	**0.292 (0.142, 0.599) < 0.001**	**0.413 (0.174, 0.979) 0.045**	**0.321 (0.133, 0.772) 0.011**

^1^Models were adjusted for age, gender, race, PIR, educational levels, smoking status, drinking status, NHANES survey cycle, BMI, total energy intake, hypertension, diabetes, CKD, CVD, and cancer.

None: no coffee consumption.

Bold represents *P*-value <0.05.

### 3.6. Sensitivity analysis

In the sensitivity analysis, an unweighted analysis was performed on the same samples to support validation of the results of the analysis using the weighting procedure. The unweighted analysis results are presented in Supplementary Tables 2–5. For all-cause mortality, similar results to the weighted analysis were observed in the unweighted analysis. However, for cardiovascular mortality, the significance of the association between decaffeinated coffee and death was reduced.

When analyzing coffee with high caffeine content (Supplementary Table 6), we found that among participants who consumed this type of coffee, those with global cognitive impairment did not exhibit an increased risk of all-cause mortality or cardiovascular death compared to those without global cognitive impairment (*P* > 0.05). At the same time, those who did not consume coffee with high caffeine content and suffered from global cognitive impairment still presented the highest risk of all-cause (HR = 2.20, 95% CI: 1.48–3.27, *P* < 0.001) and cardiovascular mortality (HR = 1.94, 95% CI: 1.05–3.60, *P* = 0.036) among all four groups.

For the joint effect of instant coffee or non-instant coffee and cognitive function on mortality (Supplementary Tables 7, 8), we found similar results as when analyzing total coffee consumption. Specifically, those who did not consume instant or non-instant coffee and suffered from cognitive impairment showed the highest risk of all-cause mortality and cardiovascular mortality.

## 4. Discussion

In this study, we investigated the associations between coffee consumption and mortality in the older US population with cognitive impairment. Our analysis found a significantly increased risk of all-cause mortality and CVD mortality in older adults with cognitive impairment and no coffee consumption compared to those with coffee consumption and normal cognitive function. In addition, when separately analyzing the older adults with cognitive impairment, we found that the association between coffee consumption and mortality was not consistent when older adults demonstrated cognitive impairment on different cognitive tests. For all-cause mortality, coffee consumption for older adults who showed cognitive impairment in the DSST or AFT showed a significant negative association with mortality and a similar association was observed in the analysis for caffeine intake from coffee. However, for older adults exhibiting cognitive impairment in CERAD-WLT, both Cox regression and RCS showed a significant association with reduced risk of all-cause mortality only when coffee consumption was located in the lower concentration interval, and for caffeine intake as well. As for global cognitive impairment, both higher coffee consumption and caffeine intake revealed a significant association with reduced risk of all-cause mortality. It was also found that all-cause mortality was not significantly increased in the global cognitive impairment group compared to the normal cognition group among those who consumed high-caffeine coffee. In addition, older adults with cognitive impairment who consumed only decaffeinated coffee displayed a significantly lower risk of all-cause mortality compared to those who did not consume coffee, which was consistent in each cognitive test. For CVD mortality, total coffee consumption revealed a significant association with mortality risk in the older adults showing cognitive impairment in DSST or AFT, with a significant decease trend in CVD mortality risk as coffee consumption increased. Whereas, coffee consumption was not significantly associated with CVD mortality in older adults who exhibited cognitive impairment in CERAD-WLT and overall cognition. In addition, for caffeine intake, we found that for people with AFT cognitive impairment, the risk of CVD mortality was significantly lower relative to those without caffeine intake only when the intake was at T2 (76.1–171.5 mg/day). For decaffeinated coffee, we found that in older adults who showed cognitive impairment in the CERAD-WLT did not display difference in the risk of CVD mortality when consuming only decaffeinated coffee compared to those who did not consume coffee.

When elucidating the associations between coffee consumption and mortality, most cohort studies and meta-analyses have revealed that coffee was beneficial in reducing mortality (5, 34, 35), while some studies also suggested that coffee consumption was not correlated with mortality or even increased the risk of mortality (28, 36). Additionally, cognitive impairment as a risk factor for mortality was extensively confirmed in a number of studies (14, 37, 38), which was consistent with the results observed in this study. In the joint effects analysis, we observed that the reduction in risk of all-cause mortality and cardiovascular mortality by coffee was most pronounced in those with cognitive impairment. This suggested that cognitive function might be an important factor influencing the uncertain relationship between coffee consumption and mortality. Although there is still a lack of cohort studies directly investigating the association between cognitive impairment, coffee consumption, and mortality, some studies investigating the effects of coffee consumption on the risk of mortality in people with neurodegenerative diseases have supported this result to some extent. One previous cohort study that investigated the associations between lifestyle factors and mortality in Parkinson’s patients found that moderate coffee consumption was associated with lower mortality in Parkinson’s patients, whereas this association was not significant in people who did not suffer from Parkinson’s disease (39). Another study about coffee consumption and mortality in Parkinson’s patients, conducted through the Cancer Prevention Study II Cohort, also determined that coffee reduced the risk of mortality (40). One animal study conducted in PGRP-LB^Δ^ -deficient fruit fly (*D. melanogaster*) to explore the pharmacological effects of caffeine on neurodegenerative diseases also found that caffeine reduced mortality of the fruit flies (41). Numerous studies have identified a possible association between coffee and neurological health. In an umbrella review including multiple observational and randomized controlled studies, higher coffee consumption was found to be associated with a reduced risk of Alzheimer’s disease (35).

Caffeine as one of the most important components of coffee has been found to have neuroprotective effects in previous clinical studies and animal studies (42–44), and a series of cross-sectional and cohort studies found that caffeine might be beneficial in improving global cognitive function (7, 8, 45). Furthermore, in addition to caffeine, coffee contains a number of other chemicals that have been found to have potential neuroprotective effects. For example, chlorogenic acid, a polyphenol abundant in both caffeinated and decaffeinated coffee. The neuroprotective effect of chlorogenic acid may be related to its ability to reduce oxidative stress. As previously found, chlorogenic acid protects some neurons from H_2_O_2_-induced oxidative damage (46, 47). Another example is trigonelline, a naturally found alkaloid in coffee beans, which has also found to be capable of ameliorating lipopolysaccharide (LPS)-mediated neuroinflammation and memory deficits in the adult mice (48). Besides, it has also been found that dietary intake of niacin, which is also one of the compounds contained in coffee, can prevent AD and age-related cognitive decline (49). Based on the associations between caffeine and a range of other compounds contained in coffee and cognition, we speculated that coffee might prevent the onset and progression of neurodegenerative diseases, such as Alzheimer’s and Parkinson’s disease, by improving cognitive function, leading to a reduction in mortality in people with cognitive impairment.

In addition, in both the AFT and DSST, coffee consumption and caffeine intake from coffee positively correlated with decreased all-cause mortality in people with cognitive impairment, whereas a decreased risk of all-cause mortality was observed only at lower concentrations of caffeine intake in the CERAD-WLT. We presumed that these variations were due to the differences in the effects of caffeine in different cognitive domains, since differences in the associations between caffeine and the results of different cognitive tests have been found in several previous studies (7, 50, 51).

For mortality caused by cardiovascular disease (CVD), the joint effect analyses of the association between cognitive impairment, coffee consumption, and mortality afforded similar results to all-cause mortality. Previous studies found that a higher cardiovascular risk was associated with lower cognitive function (52, 53), and other studies revealed the beneficial effects of coffee on reducing risk of CVD (54, 55). We hypothesized that, since people with cognitive impairment tend to have a higher risk of CVD and that coffee had a modifying effect on both cognitive impairment and CVD, coffee consumption tended to be more beneficial in people with cognitive impairment. What’s more, in specific coffee and caffeine intake and CVD mortality analyses conducted for cognitively impaired populations, a statistically significant association was also found between coffee or caffeine and mortality, but only in those who showed cognitive impairment, as measured by AFT and DSST. We speculate that possible reasons for this discrepancy are possible differences in the progression dimensions of cardiovascular disease in people with cognitive impairment in different cognitive domains, such as decreases in executive function and memory that may impair clinical communication and adherence and influence clinical decision making (56). Besides, given the strong and complex correlation between cardiovascular disease and cognitive function (57), this could also be due to the fact that people with a higher risk of developing cardiovascular disease are more likely to have impairments at the functional level as assessed by the AFT as well as the DSST.

For the analysis of decaffeinated coffee, we found that older adults who consumed only decaffeinated coffee also showed a significantly lower risk of death compared to those who did not consume coffee, suggesting that there may be chemicals other than caffeine involved in the mortality ameliorating effect of coffee on older adults with cognitive impairment. For example, the neuroprotective effects of chemicals such as niacin, trigonelline and chlorogenic acid, as well as the antioxidant, lower blood sugar and lipid-lowering functions of the phenolics and alkaloids contained in coffee (58). In some large cohort studies, decaffeinated coffee has been found to be associated with a reduced risk of all-cause or cardiovascular mortality (6, 59). In a meta-analysis, it was also found that higher decaffeinated coffee consumers showed a significant reduction in all-cause mortality compared to lower decaffeinated coffee consumers (35). Our study further validated that this association persists in cognitively impaired older adults. However, for the cognitively impaired population in the CERAD-WLT, CVD mortality were not significantly altered in the decaffeinated coffee-consuming population compared with the decaffeinated population, which we hypothesize is due to the presence of cognitively impaired people in the CERAD-WLT who are more likely to have poor prognosis for CVD, leading to the ineffectiveness of decaffeinated coffee, and of course other unconsidered confounding factors may have also contributed to this result. For the attenuated association between decaffeinated coffee and CVD death that was observed in the sensitivity analysis, this may be due to the sampling bias presented in the unweighted sample. The weighting procedure is a method of analysis applicable to the complex sampling design of NHANES, which allows the analysis to more closely match the real US population.

Our study prospectively examined the associations between coffee consumption, cognitive performance, and mortality. Although previous studies observed that moderate coffee and caffeine intake reduced all-cause and cause-specific mortality (5), this study provided a more detailed classification of the population and focused on cognitive function. Our study featured several strengths. First, we conducted the study using the NHANES database, a large sample database based on the national population, with objective evaluation criteria, strict data entry, authentic death records, and comprehensive potential confounding factors. In addition, this was the first study to explore the association between coffee intake and mortality in a cognitively impaired group, which is innovative. Finally, coffee is a staple beverage of our daily lives, and while it is still controversial whether it can be considered a healthy food, our study may provide some implications for determining health guidelines for coffee intake.

Our study also had some limitations: (1) This study specifically targeted older adults (over the age of 60) in the United States, which is not representative of the global population. (2) The dietary data provided were derived from two 24-h dietary recalls, which may not be sufficient to reflect long-term dietary habits. However, some studies suggested that two dietary recalls might be sufficient to assess daily dietary intake (60). (3) The cognitive tools that we applied only assessed some of the cognitive functions, but these tools might not be representative of overall cognitive ability. (4) Considering the short follow-up period, the incidence of specific causes of mortality was relatively low, and the associations with specific causes of death might not be observed reliably. (5) In this study we only considered coffee consumption, whereas participants may have consumed other coffee-based products (e.g., candied, cakes filled with coffee) and caffeine-rich beverages other than coffee, which may have led us to underestimate the effect of caffeine and other compounds contained in coffee for some participants.

## 5. Conclusion

In conclusion, this study found that older adults with cognitive impairment and without coffee drinking habits had a higher risk of all-cause mortality and CVD mortality compared to others. Furthermore, the association between coffee consumption and mortality differed for people with cognitive impairment occurring in different cognitive domains. We hope that this study will provide some advice for coffee consumption patterns and also provide some information for designing future clinical studies. We hope that this study will also provide some direction for future research to determine the mechanisms into how coffee affects humans.

## Data availability statement

The datasets presented in this study can be found in online repositories. The names of the repository/repositories and accession number(s) can be found below: https://www.cdc.gov/nchs/nhanes/index.htm.

## Ethics statement

Ethical review and approval was not required for the study on human participants in accordance with the local legislation and institutional requirements. The patients/participants provided their written informed consent to participate in this study.

## Author contributions

GEC, GFC, and XLW conceptualised and designed the study, critically reviewed and improved the draft manuscript, and interpreted the data. FL, YS, and XZ provided the methodology, wrote the original draft, and completed the original analyses. HW, SF, and XFW collected the data and conducted the validation. ZY searched the literature. All authors have read and approved the final manuscript.
