# Supplementary material for: Coffee consumption and all-cause and cardiovascular mortality in older adults: should we consider cognitive function?
Source: Front Nutr. 2023 Oct 24;10:1150992. doi: 10.3389/fnut.2023.1150992 (PMC10628482; doi:10.3389/fnut.2023.1150992)
Supplement: Supplementary file 1 [file Data_Sheet_1.docx]

Supplementary Material

Coffee consumption and all-cause and cardiovascular mortality in older adults: should we consider cognitive function?

**Fabin Lin ^1,2,3,4,†^, Yisen Shi ^1,2,3,†^, Xinyang Zou ^1,2,3,†^, Huaicheng Wang ^1,2,3^, Shibo Fu ^1,2,3^, Xuefei Wang ^1,2,3^, Zeqiang Yang ^1,2,3^, Guofa Cai ^5,*^ , Guoen Cai ^1,2,3,*^ and Xilin Wu ^2,*^**

^1^ Department of Neurology, Center for Cognitive Neurology, Institute of Clinical Neurology, Fujian Medical University Union Hospital, 29 Xinquan Road, Fuzhou 350001, China

^2^ Fujian Institute of Geriatrics, Fujian Medical University Union Hospital, 29 Xinquan Road, Fuzhou 350001, China

^3^ Fujian Key Laboratory of Molecular Neurology, Fujian Medical University, 88 Jiaotong Road, Fuzhou 350001, China

^4^ Department of Neurosurgery, Fujian Medical University Union Hospital, Fuzhou, 350001, China

^5^ School of Information Engineering, Guangdong University of Technology, Guangzhou, 510006, China.

*** Correspondence:**

Guofa Cai
[caiguofa2006@gdut.edu.cn](mailto:caiguofa2006@gdut.edu.cn)

Guoen Cai

[cgessmu@fjmu.edu.cn](mailto:cgessmu@fjmu.edu.cn)

Xilin Wu

xlwu@fjmu.edu.cn

**^†^**These authors have contributed equally to this work and share first authorship

# Supplementary Data

# Supplementary Figures and Tables

## Supplementary Figures


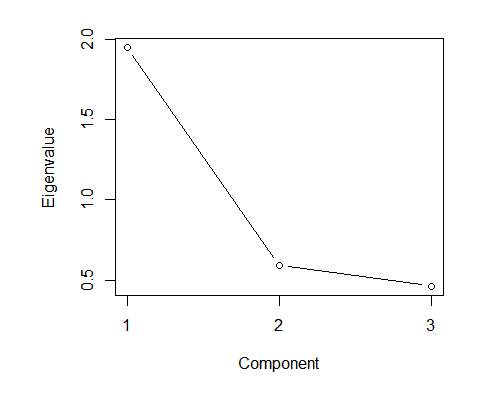


**Supplementary Figure 1.** Scree plot of eigenvalues from 3 reference cognitive tests.

|  | PC1 | PC2 | PC3 |
| --- | --- | --- | --- |
| CERAD-WLT | 0.787 | 0.546 | 0.288 |
| AFT | 0.787 | -0.543 | 0.292 |
| DSST | 0.840 | -0.002 | -0.543 |
| Proportion of variance | 0.648 | 0.198 | 0.154 |

## Supplementary Tables

**Supplement Table 1.** Loadings from a principal component analysis of 3 cognitive tests (n =2584).

|  | **All-cause mortality** | | | |
| --- | --- | --- | --- | --- |
|  | Multiple adjusted HR^1^ (95% CI), P-value | | | |
|  | Cognitive performance (CERAD-WLT) combined with total coffee intake | Cognitive performance (AFT) combined with total coffee intake | Cognitive performance (DSST)combined with total coffee intake | Cognitive performance (Global Cognition) combined with total coffee intake |
| Group1 | 1 [Reference] | 1 [Reference] | 1 [Reference] | 1 [Reference] |
| Group2 | **1.412(1.083,1.842) 0.011** | 1.259(0.969,1.637) 0.085 | 1.294(0.994,1.685) 0.055 | 1.291(0.992,1.681) 0.058 |
| Group3 | **1.391(1.107,1.749) 0.005** | **1.315(1.045,1.653) 0,019** | **1.576(1.225,2.028) <0.001** | **1.435(1.123,1.832) 0.004** |
| Group4 | **1.956(1.464,2.614) <0.001** | **2.238(1.671,2.999) <0.001** | **2.473(1.828,3.346) <0.001** | **2.258(1.680,3.035) <0.001** |
|  | **Cardiovascular mortality** | | | |
|  | Multiple adjusted HR^1^ (95% CI), P-value | | | |
|  | Cognitive performance (CERAD-WLT) combined with total coffee intake | Cognitive performance (AFT) combined with total coffee intake | Cognitive performance (DSST)combined with total coffee intake | Cognitive performance (Global Cognition) combined with total coffee intake |
| Group1 | 1 [Reference] | 1 [Reference] | 1 [Reference] | 1 [Reference] |
| Group2 | **1.622(1.016,2.591) 0.043** | 1.396(0.879,2.219) 0.158 | 1.450(0.919,2.289) 0.110 | 1.416(0.886,2.264) 0.146 |
| Group3 | **1.572(1.053,2.346) 0.027** | **1.451(0.976,2.159) 0.066** | 1.443(0.937,2.222) 0.096 | **1.556(1.019,2.375) 0.041** |
| Group4 | **2.251(1.380,3.673) 0.001** | **2.496(1.534,4.063) <0.001** | **2.401(1.431,4.027) <0.001** | **2.595(1.578,4.266) <0.001** |

**Supplement Table 2.** The joint association of cognitive impairment and total coffee consumption with all-cause mortality and cardiovascular mortality (Unweighted).

Group1: Normal cognition + had coffee consumption; Group2: Normal cognition + no coffee consumption; Group3: Cognitive impairment + had coffee consumption; Group4: Cognitive impairment + no coffee consumption.

^1^Multivariable Cox proportional hazards models were adjusted for age, sex, race, pir, educational levels, smoking status, drinking status, NHANES survey cycle, BMI, total energy intake, hypertension, diabetes, CKD, CVD, and cancer.

Bold represents P value <0.05.

|  | CERAD-WLT | AFT | DSST | Global Cognition |
| --- | --- | --- | --- | --- |
| All-cause mortality | | | | |
| Multiple adjusted HR^1^ (95% CI), P-value | | | | |
| Total coffee consumption (g/day) |  |  |  |  |
| 0 | 1 [Reference] | 1 [Reference] | 1 [Reference] | 1 [Reference] |
| 0.1 to ≤ 262.5 | 0.738(0.510,1.069) 0.108 | **0.560(0.390,0.806) 0.002** | 0.755(0.525,1.085) 0.129 | 0.700(0.485,1.010) 0.056 |
| 262.6 to ≤ 495 | 0.759(0.518,1.113) 0.158 | **0.608(0.405,0.912) 0.016** | 0.716(0.480,1.067) 0.101 | 0.761(0.514,1.125) 0.171 |
| >495 | 0.860(0.577,1.282) 0.459 | **0.469(0.301,0.729) <0.001** | **0.535(0.344,0.832) 0.005** | **0.555(0.357,0.862) 0.009** |
| P for trend | 0.598 | **0.002** | **0.006** | **0.016** |
| CVD mortality | | | | |
| Multiple adjusted HR^1^ (95% CI), P-value | | | | |
| Total coffee consumption (g/day) |  |  |  |  |
| 0 | 1 [Reference] | 1 [Reference] | 1 [Reference] | 1 [Reference] |
| 0.1 to ≤ 262.5 | 0.804(0.443,1.460) 0.474 | **0.611(0.342,1.091) 0.096** | 0.742(0.407,1.355) 0.332 | 0.760(0.425,1.359) 0.355 |
| 262.6 to ≤ 495 | 0.768(0.408,1.445) 0.414 | **0.699(0.366,1.335) 0.278** | 0.879(0.465,1.663) 0.692 | 0.741(0.390,1.407) 0.359 |
| >495 | 0.798(0.406,1.569) 0.513 | **0.308(0.136,0.694) 0.005** | **0.281(0.120,0.656) 0.003** | **0.440(0.205,0.943) 0.035** |
| P for trend | 0.540 | **0.008** | **0.006** | **0.039** |

**Supplement Table 3.** Association between coffee consumption and all-cause and cardiovascular mortality in older adults with cognitive impairment (unweighted).

^1^Model were adjusted for age, sex, race, pir, educational levels, smoking status, drinking status, NHANES survey cycle, BMI, total energy intake, hypertension, diabetes, CKD, CVD, and cancer.

Bold represents P value <0.05.

| caffeine intake from coffee (mg/day) | | | | | |
| --- | --- | --- | --- | --- | --- |
|  | 0 | 0.1 to ≤ 76 | 76.1 to ≤ 171.5 | >171.5 | P for trend |
| **All case mortality** | | | | | |
| Multiple adjusted HR^1^ (95% CI), P-value | | | | | |
|  | | | | | |
| CERAD |  |  |  |  |  |
| Cognitive impairment | 1 [Reference] | 0.693(0.480,1.002) 0.051 | 0.827(0.569,1.201) 0.318 | 0.882(0.587,1.325) 0.545 | 0.924 |
| AFT |  |  |  |  |  |
| Cognitive impairment | 1 [Reference] | **0.551(0.352,0.863) 0.009** | **0.591(0.405,0.861) 0.006** | **0.520(0.355,0.761) <0.001** | 0.058 |
| DSST |  |  |  |  |  |
| Cognitive impairment | 1 [Reference] | 0.793(0.554,1.136) 0.206 | 0.692(0.467,1.024) 0.066 | **0.509(0.324,0.799) 0.003** | **0.004** |
| Global Cognition |  |  |  |  |  |
| Cognitive impairment | 1 [Reference] | **0.551(0.348,0.873) 0.011** | 0.814(0.557,1.190) 0.289 | **0.666(0.463,0.960) 0.029** | 0.054 |
| **CVD mortality** | | | | | |
| Multiple adjusted HR^1^ (95% CI), P-value | | | | | |
| CERAD |  |  |  |  |  |
| Cognitive impairment | 1 [Reference] | 0.807(0.448,1.454) 0.475 | 0.785(0.423,1.455) 0.442 | 0.797(0.394,1.615) 0.529 | 0.613 |
| AFT |  |  |  |  |  |
| Cognitive impairment | 1 [Reference] | 0.632(0.346,1.156) 0.136 | **0.529(0.283,0.988) 0.046** | 0.456(0.207,1.005) 0.051 | 0.055 |
| DSST |  |  |  |  |  |
| Cognitive impairment | 1 [Reference] | 0.962(0.544,1.702) 0.895 | 0.665(0.347,1.274) 0.219 | **0.160(0.054,0.478) 0.001** | **<0.001** |
| Global Cognition |  |  |  |  |  |
| Cognitive impairment | 1 [Reference] | 0.814(0.461,1.436) 0.477 | 0.704(0.374,1.326) 0.277 | **0.380(0.164,0.883) 0.024** | **0.024** |

**Supplement Table 4**. Association of Caffeine intake from coffee with all-cause mortality and cardiovascular mortality in older adults presenting with cognitive impairment(unweighted).

^1^Model adjusted for age, sex, race, PIR, educational levels, smoking status, drinking status, BMI, total energy intake, hypertension, diabetes, CKD, ASCVD, cancer and NHANES survey cycle.

Bold represents P value <0.05.

|  | **Cognitive impairment presented in CERAD-WLT** | **Cognitive impairment presented in AFT** | **Cognitive impairment presented in DSST** | **Cognitive impairment presented in Global Cognition** |
| --- | --- | --- | --- | --- |
| All-cause mortality | | | | |
| Multiple adjusted HR^1^ (95% CI), P-value | | | | |
| None | 1 [Reference] | 1 [Reference] | 1 [Reference] | 1 [Reference] |
| Only intake decaffeinated coffee | **0.447(0.267,0.748) 0.002** | **0.403(0.242,0.671) <0.001** | **0.572(0.347,0.945) 0.029** | **0.452(0.274,0.746) 0.002** |
| Cardiovascular mortality | | | | |
| None | 1 [Reference] | 1 [Reference] | 1 [Reference] | 1 [Reference] |
| Only intake decaffeinated coffee | 0.540(0.244,1.196) 0.129 | 0.451(0.197,1.031) 0.059 | 0.586(0.264,1.305) 0.191 | 0.485(0.221,1.067) 0.072 |

**Supplement Table 5**. Association of decaffeinated coffee consumption with all-cause mortality and cardiovascular mortality in older adults presenting with cognitive impairment(unweighted).

^1^Model adjusted for age, sex, race, PIR, educational levels, smoking status, drinking status, BMI, total energy intake, hypertension, diabetes, CKD, ASCVD, cancer and NHANES survey cycle.

Bold represents P value <0.05.

|  | All-cause mortality | Cardiovascular mortality |
| --- | --- | --- |
|  | Multiple adjusted HR^1^ (95% CI), P-value | |
| Group1 | 1 [Reference] | 1 [Reference] |
| Group2 | 1.196(0.791,1.808) 0.397 | 0.836(0.420,1.664) 0.609 |
| Group3 | 1.630(0.983,2.704) 0.058 | 1.876(0.869,4.049) 0.109 |
| Group4 | **2.197(1.477,3.269) <0.001** | **1.941(1.046,3.604) 0.036** |

**Supplement Table 6.** The joint association of cognitive function (global) and coffee with high caffeine content consumption (Yes/No) with all-cause mortality and cardiovascular mortality (Weighted).

Group1: Normal cognition + had high-caffeine coffee consumption; Group2: Normal cognition + no high-caffeine coffee consumption; Group3: Cognitive impairment + had high-caffeine coffee consumption; Group4: Cognitive impairment + no high-caffeine coffee consumption.

^1^Multivariable Cox proportional hazards models were adjusted for age, sex, race, pir, educational levels, smoking status, drinking status, NHANES survey cycle, BMI, total energy intake, hypertension, diabetes, CKD, CVD, and cancer.

Bold represents P value <0.05.

|  | All-cause mortality | Cardiovascular mortality |
| --- | --- | --- |
|  | Multiple adjusted HR^1^ (95% CI), P-value | |
| Group1 | 1 [Reference] | 1 [Reference] |
| Group2 | 1.339(0.823,2.180) 0.240 | 1.117(0.549,2.276) 0.760 |
| Group3 | **2.185(1.307,3.651) 0.003** | **2.422(1.055,5.561) 0.037** |
| Group4 | **2.353(1.412,3.921) 0.001** | **2.442(1.206,4.944) 0.013** |

**Supplement Table 7.** The joint association of cognitive function (global) and instant coffee consumption (Yes/No) with all-cause mortality and cardiovascular mortality (Weighted).

Group1: Normal cognition + had instant coffee consumption; Group2: Normal cognition + no instant coffee consumption; Group3: Cognitive impairment + had instant coffee consumption; Group4: Cognitive impairment + no instant coffee consumption.

^1^Multivariable Cox proportional hazards models were adjusted for age, sex, race, pir, educational levels, smoking status, drinking status, NHANES survey cycle, BMI, total energy intake, hypertension, diabetes, CKD, CVD, and cancer.

Bold represents P value <0.05.

|  | All-cause mortality | Cardiovascular mortality |
| --- | --- | --- |
|  | Multiple adjusted HR^1^ (95% CI), P-value | |
| Group1 | 1 [Reference] | 1 [Reference] |
| Group2 | 1.055(0.769,1.448) 0.741 | 1.258(0.680,2.328) 0.464 |
| Group3 | **1.592(1.100,2.304) 0.014** | **2.100(1.405,3.139) <0.001** |
| Group4 | **2.238(1.630,3.074) <0.001** | **2.882(1.579,5.258) <0.001** |

**Supplement Table 8.** The joint association of cognitive function (global) and non-instant coffee consumption (Yes/No) with all-cause mortality and cardiovascular mortality (Weighted).

Group1: Normal cognition + had non-instant coffee consumption; Group2: Normal cognition + no non-instant coffee consumption; Group3: Cognitive impairment + had non-instant coffee consumption; Group4: Cognitive impairment + no non-instant coffee consumption.

^1^Multivariable Cox proportional hazards models were adjusted for age, sex, race, pir, educational levels, smoking status, drinking status, NHANES survey cycle, BMI, total energy intake, hypertension, diabetes, CKD, CVD, and cancer.

Bold represents P value <0.05.
